# Genetic Diversity and Genome Wide Association Study of β-Glucan Content in Tetraploid Wheat Grains

**DOI:** 10.1371/journal.pone.0152590

**Published:** 2016-04-05

**Authors:** Ilaria Marcotuli, Kelly Houston, Julian G. Schwerdt, Robbie Waugh, Geoffrey B. Fincher, Rachel A. Burton, Antonio Blanco, Agata Gadaleta

**Affiliations:** 1 Department of Soil, Plant and Food Sciences, Section of Genetics and Plant Breeding, University of Bari ‘Aldo Moro’, Via G. Amendola 165/A, 70126, Bari, Italy; 2 The James Hutton Institute, Invergowrie, Dundee, DD2 5DA, Scotland; 3 Australian Research Council Centre of Excellence in Plant Cell Walls, School of Agriculture, Food and Wine, University of Adelaide, Waite Campus, Glen Osmond, SA 5064, Australia; 4 Agricultural and Environmental Science, University of Bari ‘Aldo Moro’, Via G. Amendola 165/A, 70126, Bari, Italy; Institute for Sustainable Agriculture (IAS-CSIC), SPAIN

## Abstract

Non-starch polysaccharides (NSPs) have many health benefits, including immunomodulatory activity, lowering serum cholesterol, a faecal bulking effect, enhanced absorption of certain minerals, prebiotic effects and the amelioration of type II diabetes. The principal components of the NSP in cereal grains are (1,3;1,4)-β-glucans and arabinoxylans. Although (1,3;1,4)-β-glucan (hereafter called β-glucan) is not the most representative component of wheat cell walls, it is one of the most important types of soluble fibre in terms of its proven beneficial effects on human health. In the present work we explored the genetic variability of β-glucan content in grains from a tetraploid wheat collection that had been genotyped with a 90k-iSelect array, and combined this data to carry out an association analysis. The β-glucan content, expressed as a percentage w/w of grain dry weight, ranged from 0.18% to 0.89% across the collection. Our analysis identified seven genomic regions associated with β-glucan, located on chromosomes 1A, 2A (two), 2B, 5B and 7A (two), confirming the quantitative nature of this trait. Analysis of marker trait associations (MTAs) in syntenic regions of several grass species revealed putative candidate genes that might influence β-glucan levels in the endosperm, possibly via their participation in carbon partitioning. These include the glycosyl hydrolases endo-β-(1,4)-glucanase (cellulase), β-amylase, (1,4)-β-xylan endohydrolase, xylanase inhibitor protein I, isoamylase and the glycosyl transferase starch synthase II.

## Introduction

Fermentable complex polysaccharides, namely dietary fibres, from the cell walls of the pericarp-seed coat, starchy endosperm and aleurone layers of wheat (*Triticum aestivum*), barley (*Hordeum vulgare*), and oat (*Avena sativa*) grains, can lower the risk of serious, diet-related chronic diseases [1]. A large clinical study called the European Prospective Investigation into Cancer and Nutrition (EPIC) showed that dietary fibre consumption lowers the risk of colon cancer and diverticular diseases [2]. A diet that regularly contains prescribed quantities of fibre can reduce serum cholesterol and glucose levels, and thus the risk of obesity, type II diabetes and cardio-vascular disease (CVD) [3,4].

The most important fibre components in cereal grains are β-glucans and arabinoxylans, which are strongly linked to food digestibility, bulking and fermentability due to their structural properties. The viscosity of oat β-glucan is 9.6 dlg-1, barley and wheat are around 5 dlg-1, with arabinoxylan from wheat displaying a lower viscosity of 0.8–5.5dl.g-1 [5]. These polysaccharides increase the viscosity of the contents of the small intestine and therefore slow down enzyme-mediated starch breakdown. This prolongs food absorption and slows the rate of glucose release after meals, reducing the glycaemic index and therefore benefiting people with type II diabetes [6]. In the large intestine, these dietary fibres are fermented to various short-chain fatty acids that are suggested to reduce the risk of colorectal cancer [7]. The dietary fibres also have a significant impact on the health of microbial flora in the human gut and are recognized as potential pharmaceutical preventative agents of diet-related chronic diseases, when taken at appropriate doses.

The β-glucans are abundant in cell walls of Poaceae and represent one example in which heterogeneity in fine structure is essential for function in the plant cell wall [8]. β-glucans are unsubstituted, unbranched polysaccharides of β-D-glucopyranosyl monomers polymerized through (1,3)- and (1,4)-linkages, which separate the polysaccharide predominantly into cellotriosyl and cellotetraosyl units. The ratio of these, referred to as the DP3:DP4 ratio (degree of polymerisation) provides information about the fine structure and physicochemical properties of the polysaccharide. Within the grasses, barley (*Hordeum vulgare*), oat (*Avena sativa*) and rye (*Secale cereale*) grain are high sources of β-glucans, while wheat (*Triticum aestivum*), rice (*Oryza sativa*) and maize (*Zea mays*) contain lower concentrations of this polysaccharide [9]. The DP3:DP4 ratio is a useful predictor of the relative solubility of the β-glucan, and this property also varies between these species. In terms of increased solubility, the typical range of DP ratios for several cereal crops are as follows; in wheat the typical range is from 3.0:1 to 4.5:1; in barley from 2.9:1 to 3.4:1; in rye about 2.7:1; and in oats from 1.8:1 to 2.3:1 [10]. The result of this linkage arrangement is an irregular conformation of the chain. For this reason the molecules do not align over extended regions and remain in solution [11]. Furthermore, the asymmetrical shape is presumed to be responsible for the high viscosity of β-glucans, and is consequently directly related to beneficial effects on human health and nutrition [12].

As for many other plant polysaccharides, only limited information is available as to how and where β-glucans are synthesised, which genes are involved and the specific functions, interactions and activities of each protein. However, gene families involved in the synthesis of these polysaccharides have been identified and include the *cellulose-synthase-like* (*Csl*) genes [13]. Models of the intercellular location of the synthesis and assembly of β-glucan have been proposed [14,15]. Quantitative trait loci (QTL) analysis of genes controlling mature barley grain β-glucan content, coupled with the identification of *CslF* genes in a syntenic region of the rice genome, was the first step towards isolating the genes involved in β-glucan biosynthesis [16]. Subsequently, Arabidopsis, which is a dicot devoid of β-glucans was transformed with rice *OsCslF* genes in gain-of-function experiments. When expressing certain *CslF* genes, β-glucan was produced and deposited in the cell walls as shown by immunocytochemical and enzymatic methods, thus indicating that *CslF* genes are essential for β-glucan biosynthesis [13]. Using a similar approach was demonstrated that the *CslH* gene family has a role in β-glucan biosynthesis. In both cases, although expression was driven by the constitutive 35S promoter, only a small amount of β-glucan was synthesised and only in certain cell types. Hence, it is likely that one or more additional proteins are required to interact with CSLF and CSLH enzymes for efficient or correct synthesis to occur.

Several QTL studies of grain β-glucan content using both bi-parental and association mapping populations have been carried out in barley [16–22] and oats [23–25]. Much of the earlier work in mapping grain β-glucan content in barley focused on identifying loci that would confer low levels of grain β-glucan content for the malting and brewing industries. However, the health benefits associated with the consumption of β-glucan has prompted a search for loci contributing to increased levels of β-glucan, which are desirable for the functional food market. The studies mentioned above yielded numerous QTL linked to grain β-glucan content, some of which were consistent across the different sets of germplasm used by these authors, whilst others appeared to be restricted or unique to certain sets of germplasm. The objective of the present work was to carry out an association mapping analysis on grain β-glucan content in a collection of tetraploid wheats in order to identify regions of the wheat genome that are linked to this trait. The germplasm for analysis was chosen to maximise the potential for variation in this trait, given that wheat typically contains lower levels of total β-glucan and thus a correspondingly narrower range of variation in β-glucan content. Thus, a synteny-based approach was applied to identify candidate genes within the regions of the genome found to be associated with grain β-glucan content in durum wheats.

## Materials and Methods

### Plant material

A collection of 230 tetraploid wheat (*Triticum turgidum L*.) genotypes was grown in replicated trials in southern Italy (Valenzano, Bari) (field studies did not involve endangered or protected species and for those no permission were required) in 2012 and 2013 and characterised for grain β-glucan content. The collection included 123 cultivars of durum wheat (ssp. *durum*), 16 accessions of ssp. *turgidum*, 20 of ssp. *turanicum*, 19 of ssp. *polonicum*, 13 of ssp. *carthlicum*, 18 of ssp. *dicoccum*, 11 of ssp. *dicoccoides*, and 10 of ssp. *durum* var. *aethiopicum*. A randomized complete block design with three replications and plots consisting of 1-m rows, 30 cm apart, with 80 germinating seeds per plot, was used in the field experiments. During the growing season, 10 g of nitrogen per m^2^ was applied at the beginning of planting and standard cultivation practices were adopted. Plots were hand harvested at maturity and grain was stored at 4°C. Using the 1093 Cyclotec Sample Mill (Tecator Foss, 119 Hillerød, Denmark), the grain was ground and passed across a 0.1 micron sieve. These flour samples were stored in airtight containers.

### β-glucan analysis

The β-glucan content in wheat whole grain was assayed using the Mixed-Linkage β-glucan Assay Kit (Megazyme International Ireland Ltd, Wicklow, Ireland) based on the accepted method by McCleary and Codd [26] and included the industrial standard for barley [4.1% of β-glucan]. Each sample was analysed in duplicate in 2013.

### Statistical analysis

GenStat version14 was used to carry out an ANOVA on β-glucan content to identify how much of the variation in this trait could be attributed to genotype. Broad-sense heritability (H^2^), was estimated from variance components with the equation *H*^2^ = σ^2^_G_ / [σ^2^_G_ + (σ^2^_GE_ / E) + (σ^2^_e_ / rE)], with σ^2^_G_, the genetic variance; σ^2^_GE_, the genotype x environment interaction variance; σ^2^_e_, the residual variance; E, the number of environments (in this case 2); r, the number of replicates per line (in this case 3 replicates) [27].

### QTL and candidate gene detection

Samples were genotyped using a Wheat 90K iSelect array [17] and SNPs assigned to chromosomes as described in Marcotuli et al. [28] using the consensus map from Maccaferri et al. [29]. Prior to GWAS, markers with a minimum allele frequency of less than 10% and those that had >5% missing data points were removed from the data matrix using GenAlex [30,31].

To identify population structure, GenAlex [30,31] was used to carry out a principal coordinate analysis (PCoA) of the genotypic data obtained from the 230 tetraploid wheat lines. An unrooted Bayesian tree was constructed using the bootstrap method with MEGA software version 5.2.2 [32]. The Bayesian clustering program STRUCTURE version 2.3.4 [33–36], was applied, selecting an admixture model with correlated allele frequencies, for the number of populations (k) = 2 (twenty replicates), with a burn-in period of 10 × 1003 iterations followed by 10 × 1003 Markov chain Monte Carlo (MCMC) iterations. To determine the source of variation in grain β-glucan in the dataset, analysis of variance was carried out using a standard ANOVA (mixed model analysis of variance with genotype as fixed factor and blocks as random factor). TASSEL 5 [37] was used to identify regions of the genome with significant associations with grain β-glucan content. QTL analysis was performed for each single year and considering the β-glucan content estimated for both years as a covariate, only significant QTL in both environments were reported. We carried out the association analysis using two different models—a General Linear Model (GLM) using estimates of admixture from a Q matrix—GLM (Q), and a Mixed Linear Model (MLM) using a Q matrix and a kinship matrix (K) MLM–(Q +K). QQ plots were produced using TASSEL 5 to identify which model described the data best. A false discovery rate (FDR) with a threshold of <5% was calculated using the q-value package [38] in R version 3.1.1 [39] to provide adjusted p values. The correlation coefficient (R^2^) and the marker effect estimates for each genotypic class (homozygous or heterozygous) for each of the markers associated with β-glucan content were estimated using the software package TASSEL 3.0.

Putative candidate genes associated with β-glucan content were identified using the contig sequences from the Unité de Recherche Génomique Info (URGI) website [40] blasted against the monocot sequences annotated in NCBI [41]. Each “gene” or “gene family” name was searched for in the CAZy database [42] in order to find *Triticum* sequences. All the retrieved protein sequences were BLASTed against the Wheat 61k GeneChip annotated at the PLEXdb database [43], to identify expression data variation in experiment TA3H31–39 which includes three time points during grain development [44].

## Results

### Grain β-glucan content in tetraploid wheats

**Fig 1:**
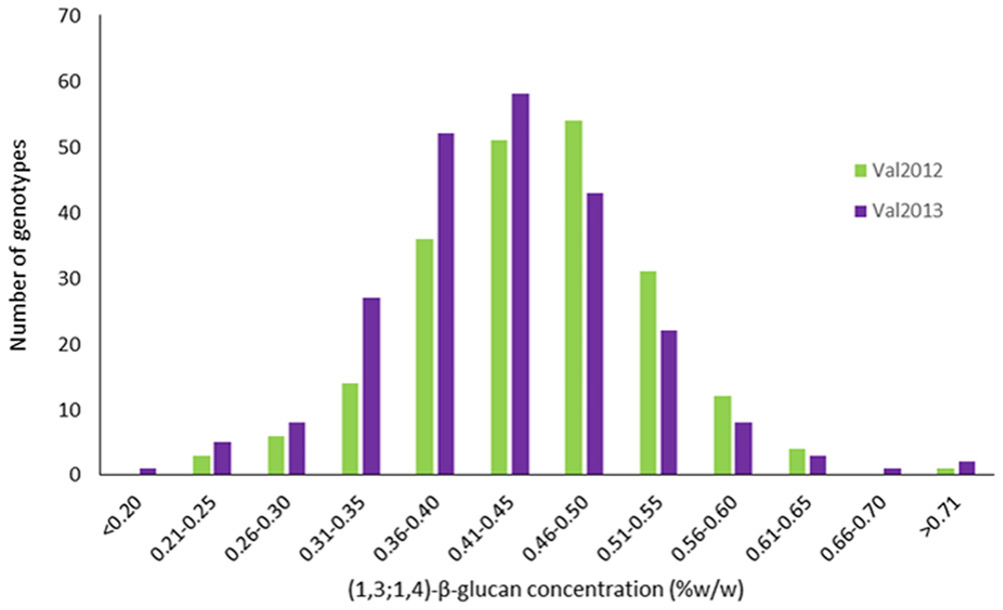
Frequency distribution of β-glucan content for 230 *Triticum turgidum* accessions grown in Valenzano 2012 and 2013.

Genetic variability for grain β-glucan content was assessed in a collection of 230 tetraploid wheat genotypes, analysed in two different years (2012 and 2013), and expressed as a percentage w/w of kernel dry weight. Variation for β-glucan content ranged from 0.18 to 0.89% for 2013 and from 0.21 to 0.71% in 2012 with a mean value of 0.42% for both environments (Table 1, Fig 1). An ANOVA test revealed highly significant variation (P≤0.001) among genotypes (Table 2). The broad sense heritability (H^2^) was 0.81 (Table 1), indicating that the phenotype was largely due to a genotypic effect. To evaluate the variability among the seven subspecies that were represented within the collection of tetraploid wheats analysed in this study, the mean values of the subgroups were compared (Table 1).

**Table 1:** Descriptive statistics of (1.3;1.4)-β-glucan content for the tetraploid wheat collection grown at Valenzano (Bari, Italy) in two years (2012 and 2013).

Sub-species	(1,3;1,4)-β-glucan (% w/w)					
	2012			2013		
	Mean	SD	Min-Max	Mean	SD	Min-Max
**Whole collection**	0.42	0.08	0.21–0.71	0.42	0.09	0.18–0.89
subsp. d*urum*	0.47	0.07	0.28–0.71	0.43	0.08	0.18–0.70
subsp. *turanicum*	0.42	0.08	0.30–0.54	0.41	0.07	0.27–0.51
subsp. *polonicum*	0.45	0.08	0.28–0.56	0.51	0.15	0.32–0.89
subsp. *turgidum*	0.40	0.10	0.21–0.52	0.42	0.08	0.31–0.56
subsp. *carthlicum*	0.42	0.05	0.35–0.52	0.36	0.09	0.23–0.52
subsp. *dicoccum*	0.41	0.06	0.32–0.51	0.38	0.05	0.29–0.46
subsp. *dicoccoides*	0.39	0.09	0.23–0.50	0.42	0.08	0.33–0.59
subsp. d*urum var*. *aethiopicum*	0.38	0.04	0.34–0.45	0.41	0.07	0.32–0.54
**s**^**2**^_**G**_				0.007		
**s**^**2**^_**P**_				0.009		
**H**^**2**^				0.80		

**Table 2:** Analysis of variance. Mean squares from the analysis of variance of (1,3;1,4)-β-glucan in the tetraploid wheat collection grown at Valenzano (Bari, Italy).

Source of variation	Degrees of freedom	Mean square
Blocks	2	0.018
Genotype	229	0.023***
Error	446	0.002

*** significant differences P≤0.001

### Molecular data and population structure

After excluding SNPs that either failed to amplify, were monomorphic, were located on the D genome or didn’t meet the criteria for missing data and allele frequency described in the methods section we had a set of 15,211 SNPs (18.6% from the original arrays) to use for genome wide association mapping. This provided us with an average marker density of 6 SNP/cM. From this subset the highest number of SNPs (8,810) were found on the B genome compared to the A genome (6,401).

**Fig 2:**
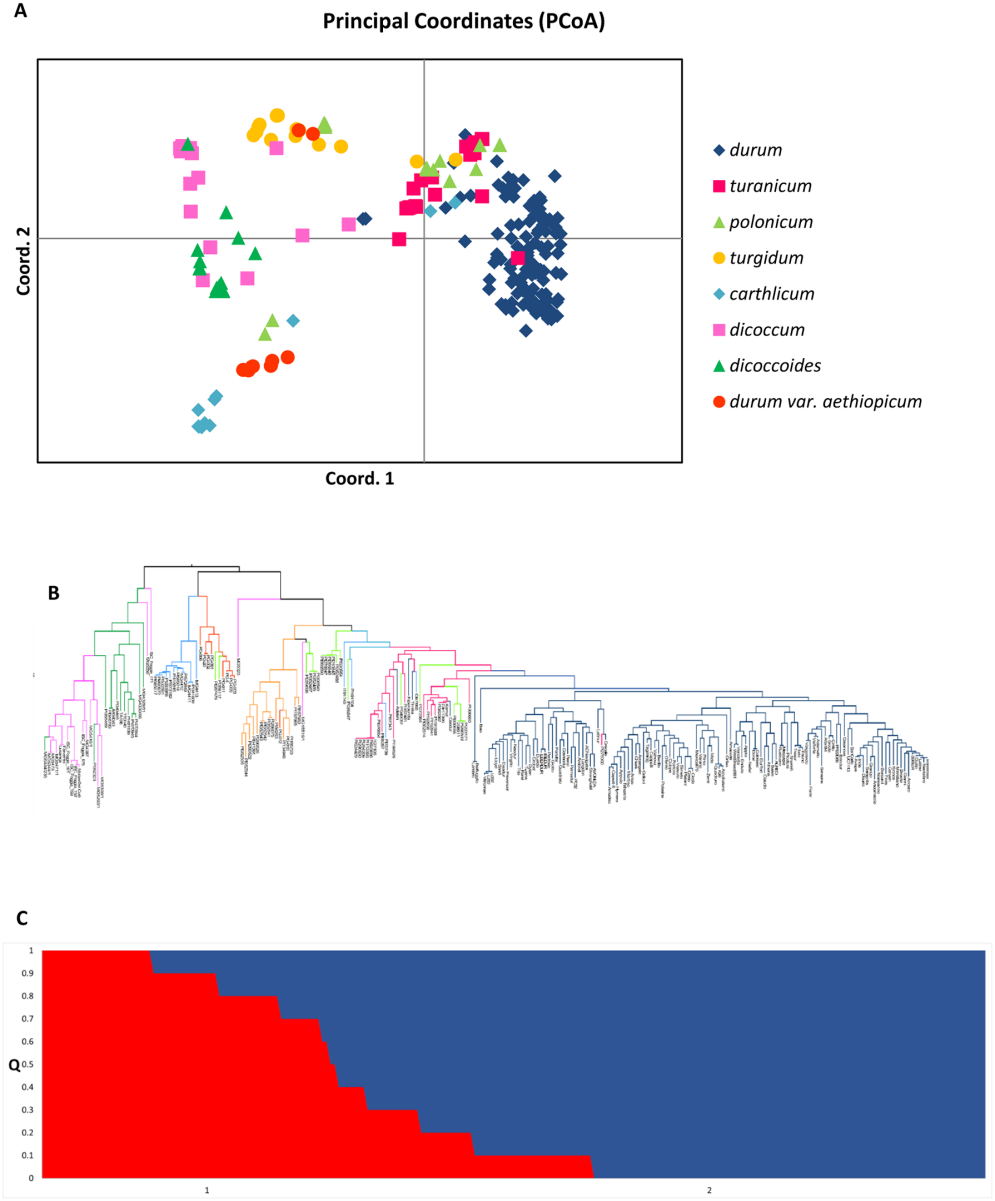
Genotypic data used to carry out a genome wide association study (GWAS). (A) Principal Coordinates Analysis (PCoA) plot of the first two components obtained from 90 K SNP iSelect for 230 tetraploid wheat accessions. (B) Unrooted bayesian tree of 90 K SNP iSelect using the bootstrap method with a replication of 1000 times. (C) STRUCTURE bar plot for K = 2 based on 90 K SNP iSelect genotyping data. Q value represents proportion of ancestry to a given subpopulation.

Principal coordinate analysis (PCoA) (Fig 2A) was carried out with the data set from 230 genotypes and 15,211 markers in order to identify genetic structure within the dataset. The first three coordinates explained 13.5%, 5.8% and 4.9% of the variation, respectively, accounting for 27.1% in total. Using the maximum likelihood method, an unrooted Bayesian tree was constructed to group accessions into clades based on the genetic data from the 90K iSelect array. This phylogenetic approach confirmed the subgroup divisions from the PCoA, showing the durum accessions clustering together, with the exception of four cultivars (Timilia, Kiperounda, Aziziah, and Ceedur) (Fig 2B). To assign individuals into subpopulations based on genetic similarity, we used a Bayesian approach implemented in STRUCTURE. Following the methodology described in Evanno et al. [45], the ΔK were plotted against the K numbers of the sub-groups. Previous work by Laidò et al. [46] indicated that for this collection of tetraploid wheats the most likely number of subpopulations is 2. Therefore considering K = 2, the collection was split in two sub-groups (group 1, group 2) containing 72 and 158 accessions based on SNP data (Fig 2C). In particular, all of the durum genotypes were assigned to cluster 1, with a Q1 mean membership of 0.92. The remaining genotypes were assigned to group 2, with a Q2 mean of 0.87.

### Analysis of marker-trait associations and QTL detection and validation

**Fig 3:**
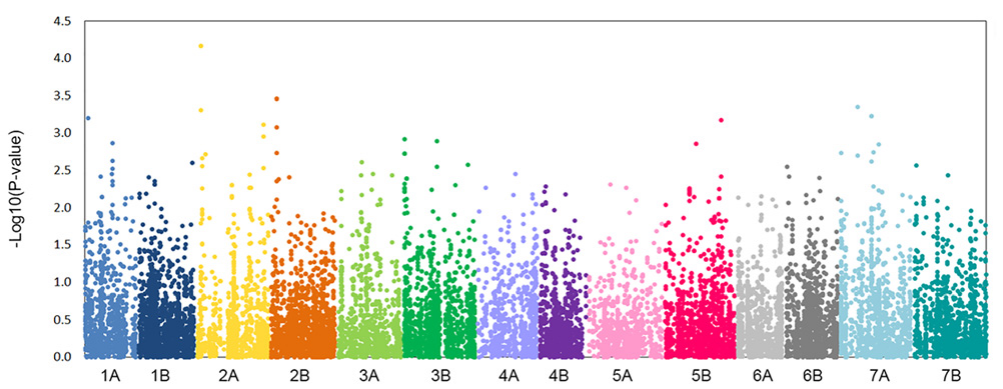
Manhattan plot of grain β-glucan content from GWAS using the Mixed Linear Model (Q+K). The -log10 (p-values) from the GWAS are plotted according to the genetic position of the SNP markers on each of the 7 wheat chromosome pairs.

The MLM (Q+K) model described the data much better than the GLM (Q) model (S1 Fig), and identified 12 marker trait associations representing seven regions associated with β-glucan content (Table 3, Fig 3). A QTL was considered significant when one or more markers were associated with β-glucan content at–Log10(P)>3. QTL linked to β-glucan content in grain were found on chromosomes 1A, 2A, 2B, 5B and 7A. The highest number of marker trait associations (MTAs) identified was on chromosome 2A (five MTAs identifying two QTL regions), followed by chromosome 7A (three MTAs identifying two QTL regions) with the lowest number on chromosomes 1A and 5B (one MTA for each QTL). More MTAs were identified on the A-genome, which had nine MTAs for five QTL, compared to the B-genome, which had three MTAs for two QTL. Two suggested QTL at the sub-threshold 2.8≤–Log10(P)≤3.0 values were identify on chromosomes 1A and 3B (Table 3). No relation were detected between *Triticum* subspecies and allelic variants and/or allelic frequencies (S1 Table).

**Table 3:** Marker-trait associations for β-glucan content.

QTL	SNP name	SNP ID	Alleles	Chr	cM	-Log10(P)	R^2^	Marker effect	Gene annotation in *Triticum* spp.	Candidate enzyme	CAZy	Monocots
*QGbg*.*mgb-1A*.*1*	Kukri_rep_c110838_253	IWB42976	A/G	1A	10.6	3.2	2.8	-0.02	-	-	-	*Z*. *mays; O*. *brachyantha; O*.*minuta*
*QGbg*.*mgb-1A*.*2*	Kukri_c43410_348	IWB45341	A/G	1A	81.6	2.8*	2.3	-0.02	*Cel9*	endo-β-1,4-glucanase	GH9	*H*. *vulgare; B*. *distachyon; S*. *italica; O*. *sativa*
*QGbg*.*mgb-2A*.*1*	Tdurum_contig10785_816	IWB66738	C/T	2A	11.2	3.3	2.7	-0.05	*WSs2A*	starch synthase II	GT5	*H*. *vulgare; S*. *bicolor; Z*. *mays; O*. *sativa; B*. *distachyon*
*QGbg*.*mgb-2A*.*2*	Excalibur_c44834_80	IWB26593	C/T	2A	197	3.1	2.6	0.02	*Bamy1*	β-amylase	GH14	*H*. *vulgare; S*. *bicolor; Z*. *mays; O*. *sativa; B*. *distachyon*
*QGbg*.*mgb-2B*	BobWhite_c25359_132	IWB1898	C/T	2B	14.5	3.5	2.9	0.07	*Wxl1*	(1,4)-beta-xylanase	GH10	*H*. *vulgare; S*. *bicolor; Z*. *mays; O*. *sativa; S*. *italica; A*. *tauschii; B*. *distachyon*
*QGbg*.*mgb-3B*	BS00091867_51	IWB11735	C/T	3B	97.2	2.9*	2.3	0.04	*Xip-II*	Xip-II gene xylanase inhibitor	GH18	*H*. *vulgare; S*. *bicolor; Z*. *mays; O*. *sativa*
*QGbg*.*mgb-5B*	Tdurum_contig35470_227	IWB70546	C/T	5B	166	3.2	2.6	-0.04	-	-	-	*O*. *sativa*
*QGbg*.*mgb-7A*.*1*	tplb0024a09_829	IWB74166	C/T	7A	49.7	3.4	2.8	0.01	-	isoamylase	GH13	*H*. *vulgare; S*. *bicolor; Z*. *mays; O*. *sativa; S*. *italica*
*QGbg*.*mgb-7A*.*2*	Tdurum_contig19352_76	IWB68797	A/G	7A	90.9	3.2	2.7	-0.03	*1-FEH*	fructan 1-exohydrolase	GH32	*H*. *vulgare; Z*. *mays; S*. *bicolor; O*. *sativa*

* Suggestive QTL at 2.8<-Log10(P)<3.0 are reported when the QTL is collinear with a putative candidate gene involved in the β-glucan biosynthetic pathway

QTL analysis was performed for each single year and considering the β-glucan content estimated for both the years as a covariate, only significant QTL in both environments were reported. For each of them, the associated SNP with the chromosome position, the -Log10(P) value, the R2, and the marker effect are reported. The enzyme families and the putative genes involved in the β-glucan biosynthetic pathway identified by using the URGI website and the Carbohydrate-Active enZYmes (CAZy) Database are also described. The syntenic relationships between *Triticum* and all the monocot gene sequences are reported in the last column.

Using the SNP sequences provided by Wang et al. [47] and the annotated contigs from the URGI database [40], we conducted a bioinformatics analysis to identify genes involved in the synthesis and breakdown of grain polysaccharides. Genes encoding glycosyl transferases (GT) or glycosyl hydrolases (GH) were located in five of the seven QTL detected. On chromosome 2A we identified starch synthase II (*WSs2A*) and β-amylase, on chromosome 2B the (1,3–1,4)-β-glucan 4-glucanohydrolase (*Glb 2*), and on chromosome 7A an isoamylase and a (1,4)-β-xylan endohydrolase (Table 3). Further investigations on MTAs under suggestive QTLs also identified an endo-(1,4)-β-glucanase (*Cel9*), or cellulose, and a xylanase inhibitor protein (*Xip-1*).

Using the Wheat 61k GeneChip [43] we carried out an *in silico* gene expression analysis which indicated that four putative candidate genes identified in our association analysis are expressed in the endosperm (Table 4). This subset of genes included an endo-(1,4)-β-glucanase, a starch synthase II, an isoamylase, and a (1,4)-β-xylan endohydrolase that encode enzymes involved in carbohydrate metabolism.

**Table 4:** Expression analysis from PLEXdb database of candidate genes influencing β-glucancontent.

Gene	Caryopsis 3–5 DAP	Embryo 22 DAP	Endosperm 22 DAP
*starch synthase II*	11.82	5.50	11.70
*β-amylase*	5.40	4.95	5.50
*(1*,*4)-beta-xylanase*	4.84	5.04	5.47
*isoamylase*	8.44	7.93	10.55
*fructan 1-exohydrolase*	8.58	9.00	10.73

RMA analysis—GeneChip TA3H32

Gene expression measurements (with RMA normalization) are reported on three developmental tissues (caryopsis, embryo and endosperm) for Chinese Spring using the Affymetrix Wheat GeneChip.

To identify the barley homologs of these putative candidate genes, a comparison between the wheat and barley genomes was conducted. Using the genomic sequences of the candidate genes highlighted by our association analysis in wheat, we searched the morexGenes database [48] to identify the barley homologs (Table 5). As expected based on previous work by Mayer et al. [49] we observed well-conserved synteny between the chromosome locations for candidate genes identified in wheat and their barley homologs. The only exception was starch synthase II which was located on 2A in wheat but its barley homolog is on 7H.

**Table 5:** Synteny between *Triticum* and *Hordeum* candidate genes involved in the β-glucan pathway. For each putative candidate gene, the chromosome position is as according to the wheat consensus map by Maccaferri et al. (2014) [29] and the Morex genome release [73].

Wheat					Barley				
Gene	Enzyme	Chrom	cM	Gene	Enzyme	Chrom	cM	Morex contig	Morex gene ID
*WSs2A*	starch synthase II	2A	11.2	*-*	starch synthase II	7H	67.6 *	contig_56779	MLOC_69670
*Bamy1*	β-amylase	2A	196.5	*Bamy1*	β-amylase	2H	55.9	contig_135953	MLOC_5168
*Wxl1*	(1,4)-beta-xylanase	2B	14.5	*X-1*	glycosyl hydrolase	2H	8.5	contig_274377	MLOC_44240
-	isoamylase	7A	49.7	*-*	isoamylase I	7H	67.3	contig_316571	MLOC_48036
*1-FEH*	fructan 1-exohydrolase	7A	90.9	*6-SFT*	fructan 6-fructosyltransferase	7H	0.5	contig_53232	MLOC_6753

* Chromosome position according to the Popseq map [74].

## Discussion

Cereals are an important source of bioactive molecules such as polysaccharides, phenolic compounds and carotenoids. Although β-glucan is a minor component of the wheat starchy endosperm cell wall, it is one of the most important portions of the fermentable fibre fraction in terms of beneficial effects for human health [12]. Despite the high nutritional value of β-glucan, few studies examining its genetic diversity have been carried out in tetraploid wheat. In our knowledge, this is the first report of QTL analysis for glucan content in durum wheat.

Here we report the phenotypic variation in β-glucan content in a collection of 230 tetraploid wheat lines. Our measurements show significant variation among the genotypes, with values comparable with previous reports [50–52]. The variation detected in grains with small seeds was from 0.32% to 1.8% (including *Triticum turgidum* and *Triticum dicoccoides*) [50]. The broad-sense heritability estimates for β-glucan content in our collection was 81%, highlighting the strong genotypic component influencing this trait in tetraploid wheats. Our broad sense heritability estimates were also in accordance with those reported for barley (82% and 88%) [53].

The dense genotyping platform utilised to provide genotypic information for our analysis, combined with increasing information becoming available from annotated genomes [54] and genetic resources such as gene expression datasets [43], enables improved prediction of candidate genes from regions underpinning the trait of interest by association analysis. A total of 12 associated markers were detected, identifying seven QTL regions associated with grain β-glucan content. The polygenic quantitative nature of this trait was confirmed by the presence of putative QTL on chromosomes 1A, 2A, 2B, 5B, and 7A. Due to the lack of any comparable studies for this trait in wheat of any ploidy level, we compared the locations of QTL we identified with studies in barley [16,18–20,22,55] and oats [23,24].

Considering that modification in SNP changes can affect different genes under one QTL, our association analysis identified putative candidate genes involved in the remodelling and hydrolysis of carbohydrate elements of the wheat grain, in particular starch. This is consistent with suggestions that there is a biochemical link between the biosynthetic pathways leading to starch and β-glucan [19,56] and further suggests that targeting genes responsible for other components of the grain, such as starch, could be an alternative way to influence the levels of β-glucan. For example, overlapping QTL for amylose and β-glucan contents was identified on chromosome 7H.

The region identified on the short arm of chromosome 2A includes a gene annotated as a starch synthase. The inverse relationship between starch and β-glucan content has been documented, particularly through the use of germplasm generated by mutagenesis. The mature grain of the M292 barley mutant, which contains a premature stop codon in *starch synthase IIa* (*SsIIa*) and was generated by sodium azide treatment of the barley cultivar Himalaya [57], has approximately 25% more β-glucan but nearly three times less starch than the parent cultivar. The *SsIIa* gene has also been shown to influence starch content in wheat, because it is responsible for elongating the (1,4)-α-glucan chains of starch [58].

Grain β-glucan and starch content have also been quantified in several mutant lines of the barley cv. Risø. The Risø13 (lys5f), Risø29 (lys5g) and Risø86 (lys5h) lines, which are allelic mutants of the *lys5* locus, contain low levels of starch but high levels of β-glucan, and in the case of lys5h this is combined with a 20% reduction in arabinoxylan content [59]. These mutations at the lys5 locus lead to increased levels of ADP-Glc in the cytosol due to a reduction in the ability of the lys5 mutants to transport ADP-Glc across the plastid envelope [60]. Another barley mutant in the same genetic background as the lys5 mutants described above but at a different locus, Risø17, has also been characterised [61]. Risø17 contains an isoamylase (*isaI*) gene that is non-functional due to an 872bp deletion in the coding region. Among other aspects of the mutant phenotype, a reduction in starch content is observed, but in this case the β-glucan content was not quantified.

We also identified an interval on the short arm of chromosome 7A that contains an isoamylase (GH13), and a β-amylase (GH14) on chromosome 2A, both of which mediate starch hydrolysis [42].

Starch comprises linear and/or slightly branched amylose and highly branched amylopectin, it is a substrate for the isoamylases enzyme, which hydrolyses the (1→6)-α-D-glucosidic branch linkages in glycogen and amylopectin influencing starch content [62,63]. Starch is also degraded by β-amylase, a member of the GH14 family of glycoside hydrolases, which catalyses the liberation of β-maltose from the non-reducing end of α-1,4-glucans, leaving a β-limit dextrin [42]. Although the two enzymes are mainly involved in transitory starch metabolism (leaf starch) they were found associated to QTL for glucan content. They may have a role in the remobilization of starch following the circadian rhythm. In fact *Arabidopsis* plants lacking β-amylase (BAM1) showed a severe starch excess phenotype associated with slower development owing to their inability to remobilize starch at night [64].

Arabinoxylan is the predominant non starch polysaccharide in the wheat endosperm cell walls, constituting 70% of these walls, compared with β-glucan, which makes up 20% [65]. Two members of the GH10 family of enzymes are putative candidate genes identified under QTL on chromosomes 2B and 7A for (1,4)-β-xylan endohydrolases. These enzymes hydrolyse (1→4)-β- xylosidic linkages in xylans [42] and therefore directly influence the amount of arabinoxylan in the grain. It is possible that they might putatively influence the β-glucan content as a percentage of total grain polysaccharide composition.

Another region identified as associated with β-glucan content in the current study included a gene encoding fructan 1-exohydrolase (*1-FEH*), which is involved in the hydrolysis of fructans through the release of terminal fructose from fructan molecules [66].

The highly conserved relationship between barley and wheat, coupled with the extensive amount of work on β-glucan content in barley, encouraged us to draw comparisons between the current findings and previous barley studies. Multiple QTL for grain β-glucan in barley have been identified, including a QTL on chromosome 2H, which is delimited by the markers Pox and ABGO19. This QTL from our analyses overlaps a QTL for α-amylase and diastatic power and therefore provides another example of a connection between starch and β-glucan levels [16]. By using consensus maps of DaRT markers, SSR, RFP and STS loci, [67] it is clear that the QTL on chromosome 2H for grain β-glucan [68] overlapped with others previously identified [16]. The current study did not identify an association with the *CslF* gene cluster on chromosome 2H, unlike Newell et al. [24] who carried out a similar analysis in oats, and several other studies in barley [16,66,20].

Due to the well-conserved synteny between barley and wheat, we were able to identify the location of the barley homologs for five putative candidate genes for grain β-glucan content in durum wheat. Except for *TtBamy1*, all homologs of these candidate genes were located in syntenous regions of the barley genome. The homolog of *TtBamy1* was positioned on the short arm of chromosome 2H rather than on the long arm like its wheat counterparts, and was near to the cluster of six *HvCslF* genes [69]. However, the wheat *CslF* homologs of the chromosome 2H cluster do not locate to the long arm, making it unlikely that this association is directly attributable to the *CslF* genes.

Studies on barley, *Arabidopsis* and rice have indicated genes from the *cellulose-synthase-like* (*Csl*) family as candidate genes for β-glucan biosynthesis, and that *CslF* and *CslH* genes are essential for β-glucan biosynthesis [13,16,70]. Furthermore, one or more additional proteins are believed to interact with CSLF and CSLH enzymes for efficient or correct synthesis to occur [13]. To date there has been no further validation of genes that have been proposed as candidates that might interact with *Csl* genes in the β-glucan synthesis pathway. Some regulatory transcription factors for cell wall genes have been identified, for example *MYB46* and secondary cell wall *CesA* genes [71], but is difficult to identify transcription factors that are putatively interacting with the *CslF* genes because there is no experimental evidence implicating any particular family of transcription factors in β-glucan synthesis.

Although the data are very interesting the candidate genes identified can only be considered to be “putative” at this stage. additional investigations should be carried out to explore the role of the putative candidate enzymes and further to identify factors involved in gene regulation (e.g. transcription factors or nucleotide interconvertors), which might play a role in determining the flux of carbon into different grain compounds.

We identified a degree of population structure within our dataset and confirmed the subgroups obtained by genotyping with SSRs and DArT markers [46], and with a subset of 104 lines from this *Triticum turgidum* collection genotyped with the same array as the current study [28]. We selected a model for our association analysis that controlled for population structure effectively illustrated by the QQ plots, and this therefore negated the potential pitfalls of carrying out an association analysis in a structured population. Ideally GWAS is carried out using a collection of germplasm with minimal structure, to avoid false positives [72], but in this case we were concerned that if we restricted the germplasm used to one subgroup there would not have been enough variation in β-glucan content within the dataset.

In conclusion, this GWAS allowed us to identify new QTL for β-glucan content in tetraploid wheat grains, and has contributed to our understanding of the genetic complexity of this important agronomic trait. It has allowed us to evaluate the phenotypic variation in β-glucan content in a collection of tetraploid wheat (*Triticum turgidum*), which is an economically important species. New putative candidate genes that could be directly or indirectly influencing β-glucan content in the grain have been identified. This can now guide future genetic studies for the validation of the role these candidate genes play in affecting the final composition of mature grain, including the β-glucan content, and could further result in the more efficient use of genetic resources in breeding programs to obtain more productive and adaptable varieties.

## Supporting Information

S1 Fig Cumulative distributions of observed P values for the General Linear Model (Q), and the Mixed Linear Model (Q + K) in the genome-wide association study in 230 wheat genotypes. A) The GLM illustrated that ignoring family relationships (K) the observed P values have a strongly nonlinear pattern, suggesting that the data are not distributed as a standard normal. B) P values from MLM appear linear, suggesting good control of potential confounders (population structure and relatedness) in the analysis.
(PDF)

S1 Table MTAs identified by GWAS analysis with the allelic variants and frequencies which have a positive or negative effect on the phenotype. (PDF)

## References

[1] Fincher GB (2009) Revolutionary times in our understanding of cell wall biosynthesis and remodeling in the grasses. Plant physiology 149: 27–37. 10.1104/pp.108.130096 19126692 PMC2613713

[2] Bingham SA, Day NE, Luben R, Ferrari P, Slimani N, Norat T et al (2003) Dietary fibre in food and protection against colorectal cancer in the European Prospective Investigation into Cancer and Nutrition (EPIC): an observational study. Lancet 361: 1496–1501. 12737858 10.1016/s0140-6736(03)13174-1

[3] American Dietetic Association (1995) Position of the American Dietetic Association: Phytochemicals and functional foods. J Am Diet Assoc 95: 493–496. 7699197 10.1016/s0002-8223(95)00130-1

[4] Australian Dietary Guidelines (2013) Canberra: National Health and Medical Research Council.

[5] Collins HM, Burton RA, Topping DL, Liao M-L, Bacic A, Fincher GB (2010) Variability in Fine Structures of Noncellulosic Cell Wall Polysaccharides from Cereal Grains: Potential Importance in Human Health and Nutrition. Cereal Chemistry 87: 272–282.

[6] Allison MH, Kerin O’D, Dallas RE, Graham GG (2004) Glycemic Index and Dietary Fiber and the Risk of Type 2 Diabetes. Diabetes Care 27: 2701–2706. 15505008 10.2337/diacare.27.11.2701

[7] Ferguson LR, Harris PJ (2003) The dietary fibre debate: more food for thought. The Lancet 361: 1487–1488. 10.1016/S0140-6736(03)13219-9 12737854

[8] Burton RA, Gidley M, Fincher GB (2010) Heterogeneity in the chemistry, structure and function of plant cell walls. Nat Chem Biol 6: 724–732. 10.1038/nchembio.439 20852610

[9] Fincher GB (2009) Exploring the evolution of (1,3;1,4)-beta-D-glucans in plant cell walls: comparative genomics can help! Current Opinion in Plant Biology 12: 140–147. 10.1016/j.pbi.2009.01.002 19168383

[10] Fincher GB, Stone BA (2004) Chemistry of non-starch polysaccharides from cereal grains Encyclopaedia of grain science: Elsevier Academic Press pp. 206–223.

[11] Woodward JR, Phillips DR, Fincher GB (1983) Water-soluble (1–3), (1–4)-β-glucans from barley (Hordeum vulgare) endosperm. I. Physicochemical properties. Carbohydrate Polymers 3: 143–156.

[12] Brennan CS, Cleary LJ (2005) The potential use of cereal (1,3;1,4)-beta-D-glucans as functional food ingredients. Journal of Cereal Science 42: 1–13.

[13] Burton RA, Wilson SM, Hrmova M, Harvey AJ, Shirley NJ, Medhurst A et al (2006) Cellulose synthase-like CslF genes mediate the synthesis of cell wall (1,3;1,4)-ß-D-glucans. Science 311: 1940–1942. 16574868 10.1126/science.1122975

[14] Wilson SM, Ho YY, Lampugnani ER, Van de Meene AM, Bain MP, Bacic A et al (2015) Determining the subcellular location of synthesis and assembly of the cell wall polysaccharide (1,3; 1,4)-beta-D-glucan in grasses. Plant Cell 27: 754–771. 10.1105/tpc.114.135970 25770111 PMC4558670

[15] Kim S-J, Zemelis S, Keegstra K, Brandizzi F (2015) The cytoplasmic localization of the catalytic site of CSLF6 supports a channeling model for the biosynthesis of mixed-linkage glucan. Plant J 81: 537–547. 10.1111/tpj.12748 25557048

[16] Han F, Ullrich S, Chirat S, Menteur S, Jestin L, Sarrafi A et al (1995) Mapping of (1,3;1,4)-beta-glucan content and beta-glucanase activity loci in barley grain and malt. TAG Theoretical and applied genetics Theoretische und angewandte Genetik 91: 921–927. 10.1007/BF00223901 24169978

[17] Molina-Cano J-L, Moralejo M, Elia M, Munoz P, Russell J, Perez-Vendrell AM et al (2007) QTL analysis of a cross between European and North American malting barleys reveals a putative candidate gene for (1,3;1,4)-beta-glucan content on chromosome 1H. Mol Breed 19: 275–284.

[18] Powell W, Caligari P, Swanston J, Jinks J (1985) Genetical investigations into (1,3;1,4)-beta-glucan content in barley. TAG Theoretical and applied genetics Theoretische und angewandte Genetik 71: 461–466. 10.1007/BF00251188 24247453

[19] Islamovic E, Obert D, Oliver R, Harrison S, Ibrahim A, Marshall JM, Stuthman DD (2013) Genetic dissection of grain beta-glucan and amylose content in barley (Hordeum vulgare L.). Mol Breeding 31: 15–25.

[20] Houston K, Russell J, Schreiber M, Halpin C, Oakey H, Washington J et al (2014) A genome wide association scan for (1,3;1,4)-beta-glucan content in the grain of contemporary 2-row Spring and Winter barleys. BMC Genomics 15: 907 10.1186/1471-2164-15-907 25326272 PMC4213503

[21] Mohammadi M, Endelman JB, Nair S, Chao S, Jones SS, Muehlbauer GJ, et al (2014) Association mapping of grain hardness, polyphenol oxidase, total phenolics, amylose content, and β-glucan in US barley breeding germplasm. Molecular Breeding 34: 1229–1243.

[22] Li J, Baga M, Rossnagel B, Legge W, Chibbar R (2008) Identification of quantitative trait loci for (1,3;1,4)-beta-glucan concentration in barley grain. J Cereal Sci 48: 647–655.

[23] Kianian SF, Phillips RL, Rines HW, Fulcher RG, Webster FH, Stuthman DD (2000) Quantitative trait loci influencing beta-glucan content in oat (*Avena sativa*, 2n = 6x = 42). Theoretical and Applied Genetics 101: 1039–1048.

[24] Newell MA, Asoro FG, Scott MP, White PJ, Beavis WD, Jannink JL (2012) Genome-wide association study for oat (*Avena sativa L*.) beta-glucan concentration using germplasm of worldwide origin. Theoretical and Applied Genetics 125: 1687–1696. 10.1007/s00122-012-1945-0 22865125

[25] Asoro FG, Newell MA, Scott MP, Beavis WD, Jannink JL (2013) Genome-wide association study for beta-glucan concentration in elite North American oat. Crop Sci 53: 542–553.

[26] McCleary BV, Codd R (1991) Measurement of (1–3),(1–4)-β-D-glucan in barley and oats: a streamlined enzymatic procedure. Journal of the Science of Food and Agriculture 55: 303–312.

[27] Nyquist WE, Baker RJ (1991) Estimation of heritability and prediction of selection response in plant populations. Crit Rev Plant Sci 10: 235–322.

[28] Marcotuli I, Houston K, Waugh R, Fincher GB, Burton RA, Blanco A, et al (2015) Genome Wide Association Mapping for Arabinoxylan Content in a Collection of Tetraploid Wheats. PLoS ONE 10: e0132787 10.1371/journal.pone.0132787 26176552 PMC4503733

[29] Maccaferri M, Ricci A, Salvi S, Milner S G, Noli E, Martelli PL, et al (2014) A high-density, SNP-based consensus map of tetraploid wheat as a bridge to integrate durum and bread wheat genomics and breeding. Plant Biotechnology Journal 1–16. 10.1111/pbi.12288 25424506

[30] Peakall ROD, Smouse PE (2006) genalex 6: genetic analysis in Excel. Population genetic software for teaching and research. Molecular Ecology Notes 6: 288–295. 10.1093/bioinformatics/bts460 PMC3463245 22820204

[31] Peakall R, Smouse PE (2012) GenAlEx 6.5: genetic analysis in Excel. Population genetic software for teaching and research—an update. Bioinformatics 28: 2537–2539. 22820204 10.1093/bioinformatics/bts460 PMC3463245

[32] Tamura KPD, Peterson N, Stecher G, Nei M, Kumar S (2011) MEGA5: Molecular Evolutionary Genetics Analysis Using Maximum Likelihood Evolutionary Distance and Maximum Parsimony Methods. Mol Biol Evol 28(10): 2731–2739. 10.1093/molbev/msr121 21546353 PMC3203626

[33] Pritchard J, Stephens M, Donnelly P (2000) Inference of population structure using multilocus genotype data. Genetics 155: 945–959. 10835412 10.1093/genetics/155.2.945 PMC1461096

[34] Falush D, Stephens M, Pritchard JK (2003) Inference of population structure using multilocus genotype data: Linked loci and correlated allele frequencies. Genetics 164. 10.1093/genetics/164.4.1567 PMC1462648 12930761

[35] Hubisz MJ, Falush D, Stephens M, Pritchard JK (2009) Inferring weak population structure with the assistance of sample group information. Molecular ecology resources 9: 1322–1332. 10.1111/j.1755-0998.2009.02591.x 21564903 PMC3518025

[36] Falush D, Stephens M, Pritchard JK (2007) Inference of population structure using multilocus genotype data: dominant markers and null alleles. Mol Ecol Notes 7: 574–578. 18784791 10.1111/j.1471-8286.2007.01758.x PMC1974779

[37] Bradbury PJ, Zhang Z, Kroon DE, Casstevens TM, Ramdoss Y, Buckler ES (2007) TASSEL: software for association mapping of complex traits in diverse samples. Bioinformatics 23: 2633–2635. 17586829 10.1093/bioinformatics/btm308

[38] Dabney A, Stores JD, Warnes GR (2010) qvalue: Q-value estimation for false discovery rate control. R package version 1380.

[39] Team RC (2012) R: A language and environment for statistical computing. R Foundation for Statistical Computing.

[40] URGI Unité de Research Génomique. Available: https://urgi.versailles.inra.fr/.

[41] NCBI. Available: http://www.ncbi.nlm.nih.gov/.

[42] Lombard V, Drula E, Coutinho PM, Henrissat B (2014) The Carbohydrate-active enzymes database (CAZy) in 2013. Nucleic acids research 42: D490–D495. 10.1093/nar/gkt1178 24270786 PMC3965031

[43] Dash S, Hong L, Wise RP, Dickerson JA (2012) PLEXdb: gene expression resources for plants and plant pathogens. Nucleic acids research 40: D1194–D1201. 10.1093/nar/gkr938 22084198 PMC3245067

[44] Schreiber AW, Sutton T, Caldo RA, Kalashyan E, Lovell B, Mayo G et al (2009) Comparative transcriptomics in the Triticeae. BMC Genomics 10: 285–285. 10.1186/1471-2164-10-285 19558723 PMC2717122

[45] Evanno G, Regnaut S, Goudet J (2005) Detecting the number of clusters of individuals using the software STRUCTURE: A simulation study. Molecular Ecology Notes 14: 2611–2620 10.1111/j.1365-294X.2005.02553.x 15969739

[46] Laidò G, Mangini G, Taranto F, Gadaleta A, Blanco A, Cattivelli L et al (2013) Genetic Diversity and Population Structure of Tetraploid Wheats (L.) Estimated by SSR, DArT and Pedigree Data. PLoS One 8: e67280 23826256 10.1371/journal.pone.0067280 PMC3694930

[47] Wang SW, Forrest D, Allen K, Chao A, Huang S, Huang BE, et al (2014) Characterization of polyploid wheat genomic diversity using a high-density 90 000 single nucleotide polymorphism array. Plant Biotechnol J 12: 787–796. 10.1111/pbi.12183 24646323 PMC4265271

[48] morexGenes. Available: https://ics.hutton.ac.uk/morexGenes/index.html.

[49] Mayer K, Martis M, Hedley P, Simkova H, Liu H, Morris JA, et al (2011) Unlocking the barley genome by chromosomal and comparative genomics. Plant Cell 23: 1249–1263. 10.1105/tpc.110.082537 21467582 PMC3101540

[50] Pritchard JR, Lawrence GJ, Larroque O, Li Z, Laidlaw HKC, Morell, Matthew K, et al (2011) A survey of β-glucan and arabinoxylan content in wheat. Journal of the Science of Food and Agriculture 91: 1298–1303. 10.1002/jsfa.4316 21469147

[51] Beresford G, Stone BA (1983) (1→3),(1→4)-β-D-glucan content of Triticum grains Journal of Cereal Science 1: 111–114.

[52] Lafiandra D, Riccardi G, Shewry PR (2014) Improving cereal grain carbohydrates for diet and health. Journal of Cereal Science 59: 312–326. 24966450 10.1016/j.jcs.2014.01.001 PMC4064937

[53] Kim H-S, Park K-G, Baek S-B, Kim J-G (2011) Inheritance of (1–3)(1–4)-beta-D-glucan content in barley (Hordeum vulgare L.). J Crop Sci Biotechnol 14: 239–245.

[54] Brenchley R, Spannagl M, Pfeifer M, Barker GLA, D'Amore R, et al (2012) Analysis of the bread wheat genome using whole-genome shotgun sequencing. Nature 491: 705–710. 10.1038/nature11650 23192148 PMC3510651

[55] Molina-Cano JL, Moralejo M, Elia M, Munoz P, Russell JR, Perez-Vendrell AM et al (2007) QTL analysis of a cross between European and North American malting barleys reveals a putative candidate gene for beta-glucan content on chromosome 1H. Molecular Breeding 19: 275–284.

[56] Burton RA, Fincher GB (2012) Current challenges in cell wall biology in the cereals and grasses. Frontiers in plant science 3. 10.3389/fpls.2012.00130 PMC3375588 22715340

[57] Clarke B, Liang R, Morell M, Bird A, Jenkins C, Li Z (2008) Gene expression in a starch synthase IIa mutant of barley: changes in the level of gene transcription and grain composition. Funct Integr Genomics 8: 211–221. 10.1007/s10142-007-0070-7 18270759

[58] Konik-Rose JT, Chanvrier H., Tan I., Halley P., Gidley M., Kosar-Hashemi B., et al (2007) Effects of starch synthase IIa gene dosage on grain, protein and starch in endosperm of wheat. Theor Appl Genet 115: 1053–1065. 17721773 10.1007/s00122-007-0631-0

[59] Christensen U, Scheller HV (2012) Regulation of (1,3;1,4)-b-D-glucan synthesis in developing endosperm of barley lys mutants. Journal of Cereal Science 55: 69–76.

[60] Patron NJ, Greber B, Fahy BF, Laurie DA, Parker ML, Denyer K (2004) The lys5 mutations of barley reveal the nature and importance of plastidial ADP-Glc transporters for starch synthesis in cereal endosperm. Plant Physiology 135: 2088–2097. 15299120 10.1104/pp.104.045203 PMC520780

[61] Burton RA, Jenner H, Carrangis L, Fahy B, Fincher GB, Hylton C, et al (2002) Starch granule initiation and growth are altered in barley mutants that lack isoamylase activity. Plant Journal 31: 97–112. 12100486 10.1046/j.1365-313x.2002.01339.x

[62] Fujita N KA, Suh D-S, Wong K-S, Jane J-L, Ozawa K, Takaiwa F, et al (2003) Antisense inhibition of isoamylase alters the structure of amylopectin and the physicochemical properties of starch in rice endosperm. Plant Cell Physiol 44: 607–618. 12826626 10.1093/pcp/pcg079

[63] Utsumi Y UC, Sawada T, Fujita N, Nakamura Y. (2011) Functional diversity of isoamylase oligomers: the ISA1 homo-oligomer is essential for amylopectin biosynthesis in rice endosperm. Plant Physiology 156. 10.1104/pp.111.173435 PMC3091037 21436381

[64] Fulton DC, Stettler M, Mettler T, Vaughan CK, Liet J, Perigio F, et al (2008) Beta-AMYLASE4, a non catalytic protein required for starch breakdown, acts upstream of three active beta-amylases in Arabidopsis chloroplasts. Plant Cell 20, 1040–1058. 10.1105/tpc.107.056507 18390594 PMC2390740

[65] Bacic A, Stone BA (1980) A (1→3)- and (1→4)-linked β-D-glucan in the endosperm of wheat. Carbohydrate Research 82: 372–377.

[66] Kooiker M, Drenth J, Glassop D, McIntyre CL, Xue GP (2013) TaMYB13-1, a R2R3 MYB transcription factor, regulates the fructan synthetic pathway and contributes to enhanced fructan accumulation in bread wheat. J Exp Bot 64: 3681–3696. 10.1093/jxb/ert205 23873993 PMC3745729

[67] Wenzl H, Wodicka B (1981) The etiology of the occurrence of callose in the tubers of stolbur-diseased potato plants. Zeitschrift fuer Pflanzenkrankheiten und Pflanzenschutz 88: 584–587.

[68] Li J BM, Rossnagel BG, Legge WG, Chibbar RN. (2008) Identification of quantitative trait loci for (1,3;1,4)-β-glucan concentration in barley grain. J Cereal Sci 48: 647–655.

[69] Burton RA, Jobling S, Harvey A, Shirley N, Mather D, Bacic A, et al (2008) The genetics and transcriptional profiles of the Cellulose Synthase-Like HvCslF gene family in Barley. Plant Physiol 146: 1821–1833. 10.1104/pp.107.114694 18258691 PMC2287353

[70] Doblin M, Pettolino F, Wilson S, Campbell R, Burton R, Fincher GB, et al (2009) A barley cellulose synthase-like CSLH gene mediates (13;14)-beta-D-glucan synthesis in transgenic Arabidopsis. Proc Natl Acad Sci U S A 106: 5996–6001. 10.1073/pnas.0902019106 19321749 PMC2667043

[71] Kim E-D, Sung S (2012) Long noncoding RNA: unveiling hidden layer of gene regulatory networks. Trends in Plant Science 17: 16–21. 10.1016/j.tplants.2011.10.008 22104407

[72] Rostoks NRL, MacKenzie K, Cardle L, Bhat PR, Roose ML, Svensson JT, et al (2006) Recent history of artificial outcrossing facilitates whole genome association mapping in elite crop varieties. Proc Natl Acad Sci U S A 103: 18656–18661. 17085595 10.1073/pnas.0606133103 PMC1693718

[73] IBSC (2012) A physical, genetic and functional sequence assembly of the barley genome. Nature 491: 711–716. 10.1038/nature11543 23075845

[74] Mascher M, Muehlbauer GJ, Rokhsar DS, Chapman J, Schmutz J, Kerrie B, et al (2013) Anchoring and ordering NGS contig assemblies by population sequencing (POPSEQ). The Plant Journal 76: 718–727. 10.1111/tpj.12319 23998490 PMC4298792

