# Supplementary figures and images for: Genetic Diversity and Genome Wide Association Study of β-Glucan Content in Tetraploid Wheat Grains
Source: PLoS One. 2016 Apr 5;11(4):e0152590. doi: 10.1371/journal.pone.0152590 (PMC4821454; doi:10.1371/journal.pone.0152590)

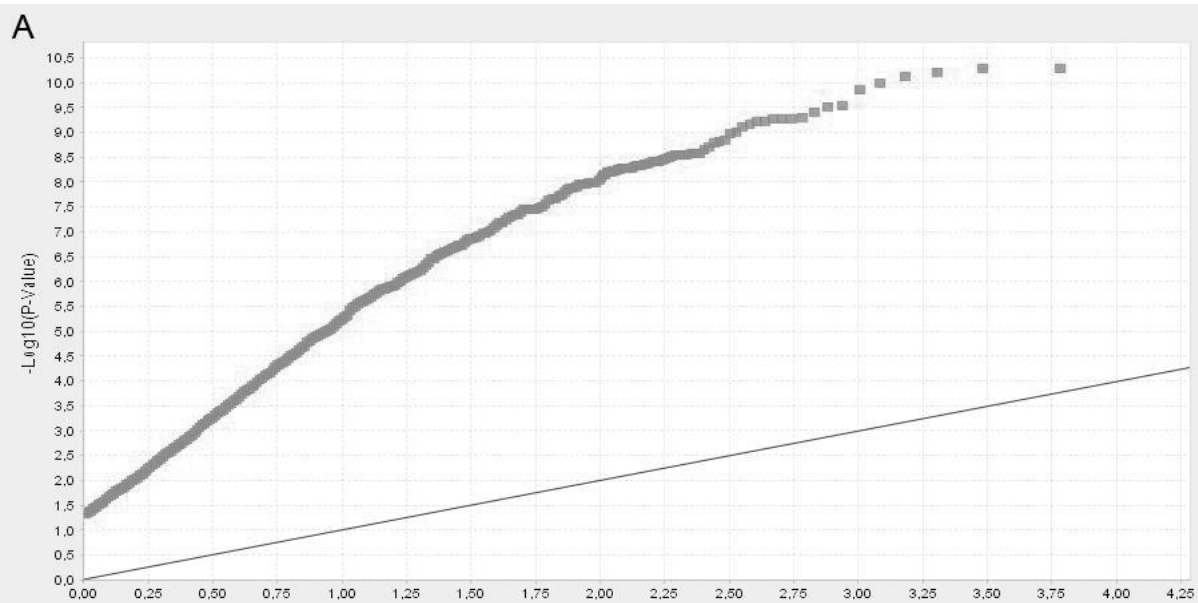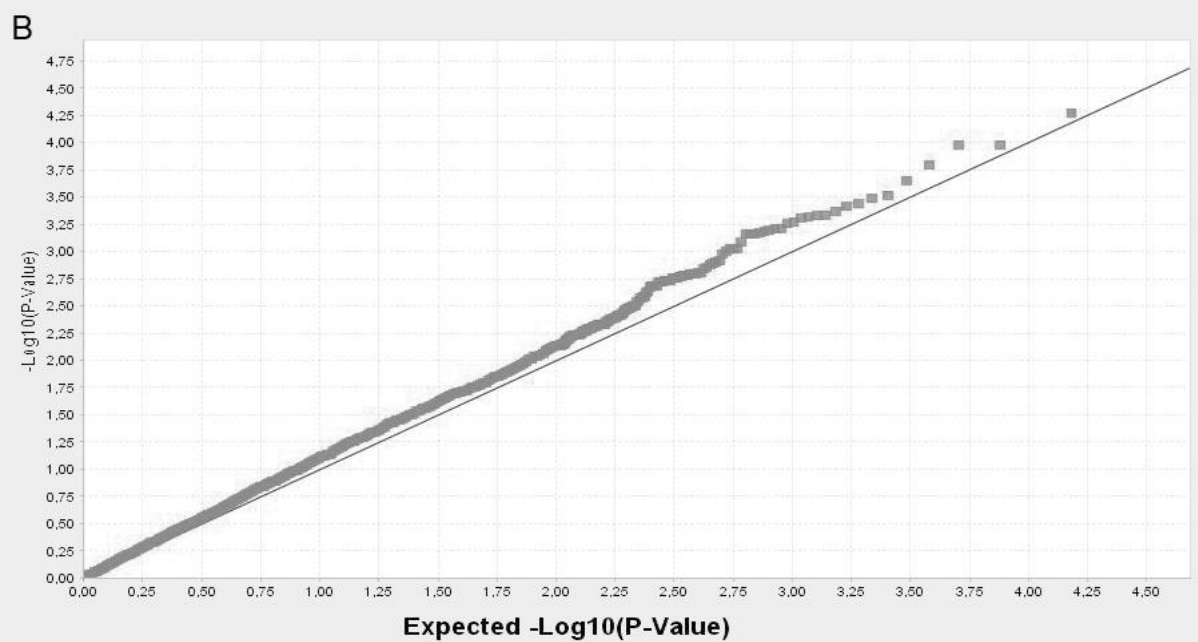

Supplement: S1 Fig — A) The GLM illustrated that ignoring family relationships (K) the observed P values have a strongly nonlinear pattern, suggesting that the data are not distributed as a standard normal. B) P values from MLM appear linear, suggesting good control of potential confounders (population structure and relatedness) in the analysis. (PDF) [file pone.0152590.s001.pdf]
