# Supplementary material for: Genetic Diversity and Genome Wide Association Study of β-Glucan Content in Tetraploid Wheat Grains
Source: PLoS One. 2016 Apr 5;11(4):e0152590. doi: 10.1371/journal.pone.0152590 (PMC4821454; doi:10.1371/journal.pone.0152590)
Supplement: S1 Table — (PDF) [file pone.0152590.s002.pdf]

SNP	Allele	Allele frequencies							
		ssp. durum	*ssp. Turanicum*	*ssp. polonicum*	*ssp. turgidum*	*ssp. carthlicum*	*ssp. dicoccum*	*ssp. dicoccoides*	*ssp. aethiopicum*
IWB42976	A	32%	50%	21%	36%	36%	22%	50%	44%
IWB45341	A	96%	100%	95%	100%	31%	28%	46%	90%
IWB66738	C	44%	95%	84%	100%	100%	94%	100%	100%
IWB26593	C	77%	11%	83%	75%	0%	59%	31%	20%
IWB1898	C	95%	90%	89%	81%	14%	61%	15%	10%
IWB11735	C	98%	100%	95%	88%	86%	33%	31%	100%
IWB70546	C	36%	5%	5%	25%	0%	0%	0%	80%
IWB74166	C	66%	80%	95%	13%	14%	50%	23%	50%
IWB68797	A	44%	65%	6%	88%	8%	100%	100%	60%
